# Beyond salt tolerance: SOS1-13’s pivotal role in regulating the immune response to *Fusarium oxysporum* in *Solanum phureja*

**DOI:** 10.3389/fpls.2025.1553348

**Published:** 2025-03-06

**Authors:** Liqin Liang, Xiaona Liu, Liuyan Guo, Liyan Wang, Yuehua Zhao, Yue Wu, Yiqian Chen, Weizhong Liu, Gang Gao

**Affiliations:** College of Life Science, Shanxi Normal University, Taiyuan, China

**Keywords:** potato, SOS1, resistance, *Fusarium oxysporum*, stomata movement

## Abstract

**Introduction:**

*Fusarium oxysporum (FOX)* causes severe Fusarium wilt in the potato (*Solanum tuberosum* group *Phureja*) annually around the world. As an Na^+^/H^+^ antiporter, SOS1, a member of the salt oversensitive (SOS) signaling pathway plays important role in salt tolerance, but its function in plant disease resistance has been less studied.

**Methods:**

The function of the potato *SOS1* gene (*StSOS1-13*) responding to the *FOX* infection was researched by gain- and loss-of-function assays.

**Results:**

StSOS1-13-overexpressed Arabidopsis differed from WT plants in multiple aspects post-*FOX* infection. It exhibited less ROS accumulation and cell necrosis in leaves, higher SOD and CAT activities accompanied by reduced MDA content, enhanced root development, increased tolerance to *FOX* infection, and an accelerated leaf stomatal closure rate along with a reduced stomatal aperture area. Additionally, the ectopic overexpression of *StSOS1-13* in Arabidopsis induced down-regulation of *AtPR12*. Conversely, silencing the ortholog gene *NbSOS1-13* in *Nicotiana benthamiana* showed more accumulation of ROS, serious cell necrosis, reduced activities of SOD and CAT, significantly increased MDA level, obvious leaf wilting, decreased tolerance to infection, and reduced leaf stomatal closure rate and accelerated stomatal area. Furthermore, the expression of SA and JA response-related genes (*NbPR5* and *NbPR12*) was up-regulated in *NbSOS1-13*-silenced plants.

**Discussion:**

These findings suggest that StSOS1-13 may serve as a key hub in the immune response to FOX infection by enhancing the antioxidant defense system, promoting root development to improve water uptake, facilitating leaf stomatal closure to minimize water loss through evaporation, and associating with the SA and JA signaling pathways.

## 1 Introduction

Plants have evolved several strategies to cope with salinity, in which the salt-overly-sensitive (SOS) pathway controls the net uptake of sodium by roots and the xylematic transfer to shoots in vascular plants (Gámez-Arjona et al., 2024; Villalta et al., 2021). SOS conduction pathway is consisted of three components: SOS1, SOS2 and SOS3 and, mediate salt stress signal transduction, transport excess Na^+^ out of the cell to maintain the relative balance of ion in the cell (Cheng et al., 2019; Jiang et al., 2019; Yang and Guo, 2017). The SOS1s of glycophytes and halophytes, located in the plasma membrane and expressed in the wood parenchyma and root epidermal cells, are primarily involved in the efflux of Na^+^ from the cytoplasm to the soil and the transport of Na^+^ to the leaves via transpiration (Ali et al., 2021).

At present, the involvement of SOS1 in plant salt tolerance has been demonstrated in a variety of plants, such as Arabidopsis (Shi et al., 2003), tomato (Olias et al., 2009), sweetpotato (Gao et al., 2012), cotton (Chen et al., 2017), soybean (Zhang M, et al., 2022; Zhao et al., 2016), maize (Zhou et al., 2022), potato (Liang et al., 2023). However, the involvement of SOS1 in plant disease resistance was only found in the immunity of *Brassica juncea* var. *tumida* SOS1 (*BjSOS1*) to *Plasmodiophora Brassicae* (Cheng et al., 2019). Furthermore, NHX1 is involved in the resistance to *Phytophthora parasitica nicotianae* (*Ppn*) in *Nicotiana benthamiana*. *NbNHX1* silencing led to the reduction of H^+^ efflux from vacuole to cytoplasts, a lower pH in vacuole, the NAD(P) (H) pool decreased, and a lower reactive oxidative species (ROS) level in cell, down-regulated of ROS-responsive genes, impaired ability to scavenge ROS induced by the pathogen, and decreased *Ppn* resistance in *N. benthamiana* (Chen et al., 2014). In contrast, transient overexpressed *NbNHX1* led to the increase of vacuolar pH and cellular ROS level in the *N. benthamiana*, which was associated with an enlarged NAD(P) (H) pool and up-regulated ROS-responsive genes, and *SeNHX1* (from *Salicornia europaea*) or *AtNHX1* (from Arabidopsis) ectopic expression enhanced the resistance to *Ppn* with a lower H_2_O_2_ concentration and the reduced blight area in the leaves. It has been shown that NHX1 is involved in plant disease defense by regulating the pH of the vacuole, affecting the oxidation state of cells, and priming the antioxidant system associated with resistance to Ppn in *N. benthamiana* (Chen et al., 2015). Nevertheless, whether SOS1 is involved in disease resistance in other plants remains to be explored further.

Potato is a significant staple crop globally, serving as an essential food source (Munthali et al., 2022) and after maize (*Zea mays*), wheat (*Triticum aestivum*) and rice (*Oryza sativa*) (Qin et al., 2022). However, the ever-changing biotic stresses are causing widespread declines in potato yields and quality (Dahal et al., 2019), such as the late blight pathogen *Phytophthora* (Ali et al., 2014), bacterial wilt pathogen *Ralstonia solanacearum* (Wullings et al., 1998) and Fusarium wilt pathogen *Fusarium oxysporum* f. *sp* (Li L, et al., 2022). Fusarium wilt disease poses a serious threat to global potato yields during the potato growing season (Li M, et al., 2022). Therefore, it is of great importance to mine stress-resistant genes at the molecular genetic level for the utilization of potato germplasm resources and the improvement of potato varieties (He F, et al., 2022). We have shown previously that the potato *SOS1* (*StSOS1-13*) gene would be potential candidate gene for potato salt-tolerant seeding (Liang et al., 2023). However, the role of SOS1 in plant disease resistance response conjugating with operation of the SOS pathway for salt tolerance has not been assessed.

In this work, we aimed to determine the precise role of StSOS1-13 in response to *FOX* using gain- and loss-of-function assays in Arabidopsis and *N. benthamiana*. This approach allows the quick and accurate dissection of the gene function and pathway constituents in simplified gene expression systems for a preliminary investigation into its molecular mechanism in *FOX* resistance through silencing orthologous gene in tobacco and heterologous overexpression in Arabidopsis. Our results demonstrate that StSOS1-13 plays a special comprehensive regulatory role in plant immunity against *FOX* infection, providing interesting insights into understanding SOS1-mediated plant disease resistance responses.

## 2 Materials and methods

### 2.1 Plant material, growth conditions and *Fusarium oxysporum* inoculation

The potato (*Solanum tuberosum* Group *phureja*), *Nicotiana benthamiana* and *Arabidopsis thaliana* (Col-0) seeds used in this study were all provided by institute of vegetables and flowers, Chinese academy of agricultural sciences, the corresponding plants were grown in a growth chamber with a 16:8 light:dark cycle, and at a 26°C/18°C: 60-70% (Ali et al., 2014), 24°C/22°C: 40% (Murphy et al., 2018) and 22°C: 70-80% (Zhang L, et al., 2022) temperature(day/night): relative humidity correspondingly. The *FOX* strain used in this study was stored in our laboratory and cultured with PDA at 28°C, and then transferred to PDB liquid culture in a shaker for propagation culture (Li M. et al., 2022), the culture solution was filtered, conidia were collected, and diluted to 1×10^7^/mL. Plant seedlings were inoculated with suspension of *FOX* with a concentration of 10^7^ spores/mL for 30 min before being transplanted back into the soil for normal culture using root infection method according to previous descriptions (Qian et al., 2022). Samples for RNA extraction were collected from the whole leaves at 6, 12, 24, 36, 48, 60, and 72 h (0 h as a control) after inoculation (Wang X, et al., 2022).

### 2.2 Vector construction and plant transformation

The tobacco rattle virus (TRV) system was used for VIGS analysis. A 300 bp interference fragment from *NbSOS1-13* (NCBI accession number: Niben101Scf00485g02023.1, the ortholog of *StSOS1-13* in *N. benthamiana*) was inserted into the pTRV2 vector by double-enzyme digestion (*Xba*I/*Bam*HI) to generate the pTRV2-*NbSOS1-13* vector and verified by sequencing (Kang et al., 2021). *NbPDS* gene (**Supplementary Note 1**) was also inserted into the pTRV2 vector and used as the positive control. The pTRV2, pTRV2-*PDS*, and pTRV2-*NbSOS1-13* vectors were transformed into Agrobacterium strain GV3101 for Agrobacterium-mediated transient transformation of plants *via* syringe infiltration (Li et al., 2021; Yin et al., 2022).

To investigate the role of *StSOS1-13* in Arabidopsis against *FOX* infection, *StSOS1-13* was overexpressed in Arabidopsis under the control of the CaMV35S. Firstly, full-length *StSOS1-13* cDNA sequence (1734 bp) was amplified using gene-specific primers, and then inserted into the pCAMBIAsuper1300 vector containing the *Xba*I and *Sac*I restriction site, and then the pCAMBIAsuper1300-*StSOS1-13* vector generated were transformed into *E.coli* DH5α and verified by sequencing (Shi et al., 2022). Secondly, the pCAMBIAsuper1300-*StSOS1-13* vectors were transformed into the *A. tumefaciens* strain GV3101 (re-suspended with a suspension containing 5% sucrose and 0.05% Silwet L-77 to OD_630_ = 0.8) using the freeze-thaw method, and then transferred into the Arabidopsis via the flower dipping method. Thirdly, the Arabidopsis plants were incubated in the dark for 24 h (Sakata et al., 2022) before being transferred to a culture chamber for normal culture. The primary seeds harvested were recorded as the T0 generation and sown on soil, the resulting plants (T1 generation) were used to screen for homozygous transgenic lines by MS medium containing hygromycin (25 mg/L). Subsequently, Hyg-resistant T2 generation Arabidopsis plants were used for further validation whether the positive plant material, including DNA extracting by a DNA Quick Plant System (TransGen Biotech, Beijing, China), and PCR analysis based on the 35S promoter using specific primers (**Supplementary Table S1**).

### 2.3 RNA extraction and RT-qPCR analysis

Total RNAs were extracted using *TransZol* Up Plus RNA Kit (Transgen, Beijing, China) and then employed as a template with *TransScript* ^®^ One-Step gDNA Removal and cDNA Synthesis Super Mix for qPCR (Transgen, China) for the first strand cDNA synthesis. The RT-qPCR was performed on the QuantStudio-3 system (Thermo Fisher Scientific, Shanghai, China). The reaction mix contained 2 µL template cDNA, 0.4 µL F primer (10 µM), 0.4 µL R primer (10 µM), 0.4 µL passive reference dye (50×), 10 µL 2×*PerfectStart* ^®^ Green Super Mix and 6.8 µL nuclease-free water. The RT-qPCR amplification parameters were as follows: predenaturation at 94°C for 30 s, followed by 35 cycles of denaturation at 94°C for 30 s and annealing at 60°C for 30 s. Finally, the relative gene expression level was calculated using the 2^−ΔΔCT^ method (He J, et al., 2022). As a reference, the *actin* of *N. benthamiana*, Arabidopsis and other primers (**Supplementary Table S1**) used in this study were designed by Primer Blast website^1^ of NCBI. Three biological experiments with three technical replicates were performed for each reaction.

### 2.4 Hydrogen peroxide localization and necrotic cells detection

The accumulation of H_2_O_2_ in the tobacco and Arabidopsis leaf tissues was visualized by 3,3′-diaminobenzidine (DAB) staining. The leaves immersed in DAB were incubated for 6 h with gentle shaking in dark conditions and the ROS fluorescence intensity was measured using ImageJ software (Yin et al., 2022).

Tobacco and Arabidopsis leaf tissue were evaluated for cell death using trypan blue staining. The leaves immersed in 0.4% trypan blue were incubated for 6 h with gentle shaking and cell death was measured using ImageJ software (Wang W, et al., 2022).

### 2.5 Biochemical analysis

The malondialdehyde (MDA) content, catalase (CAT) and superoxide dismutase (SOD) activities of tobacco and Arabidopsis tissue were determined separately using the corresponding kits, following the manufacturer’s instructions (Nanjing Jiancheng Bioengineering Institute, China).

### 2.6 Determination of fungal biomass in leaves of plants infected with *FOX*

Evaluation of *FOX* colonization in the inoculated plant leaves was determined using plate counts at specified time points by the colony forming unit (CFU) per gram of leaf tissues according to previous descriptions (Qian et al., 2022).

### 2.7 The stomatal movement observation and measurement

The stomatal movement of WT, OE5 and OE6 Arabidopsis lines were observed under a microscope (40 ×) before and after infection with *FOX*. Ten random visual fields for each group of plant and three duplicates for each line were also observed. Stomatal apertures (Stomata aspect ratios) in the images were measured using the software ImageJ (Zhu et al., 2021). The length of a stoma is defined as the distance between the internal contact points of the two guard cells, while the width is the maximum distance between the two innermost guard cells.

### 2.8 Statistical analysis

The data were presented as means ± standard deviation (SD) of at least three independent experiments. All statistical analysis was carried out by GraphPad Prism 9 software, and the significance of differences between different groups was evaluated by Student t-test or ANOVA.

## 3 Results

### 3.1 RT-qPCR analysis of *StSOS1-13* gene expression under *FOX* infection

**Figure 1:**
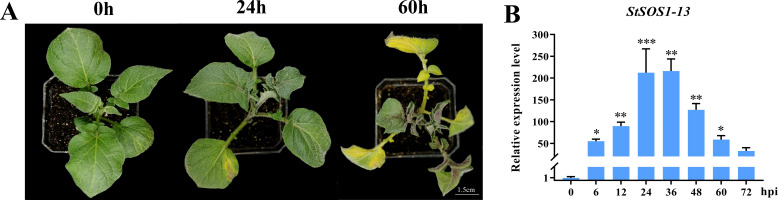
The expression analysis of *StSOS1-13* gene under *FOX* infection. **(A)** The typical withering phenotype appeared at 60 hpi; **(B)** RT-qPCR level of *StSOS1-13* gene expression under *FOX* infection. The expression level of *StSOS1-13* (with control at 0 h) was normalized against *StAct*. The vertical bars indicate the standard error of the mean. Asterisks indicate a significant difference based on the T test. (*, p<0.05, **, p<0.01, ***, p<0.001).

To investigate whether *StSOS1* genes were involved in immune response to *FOX* (**Supplementary Figure S1**), the phenotypes and *StSOS1-13* gene expression of the potato infected by root infection method was observed and analyzed. The results showed that the leaves of potato plants exhibited slight wilting symptoms at 24 hpi after inoculation with *FOX*, while exhibited severe disease symptom at 60 hpi (**Figure 1A**). The *StSOS1-13* gene expression level showed a significant trend of first increasing and then decreasing (**Figure 1B**), and rose 184-fold at 36 h after inoculation with *FOX*, suggesting that the StSOS1-13 was involved in the resistance of potato to *FOX* and played an important role.

### 3.2 Silencing of *NbSOS1-13* affected tobacco against *FOX*

To better understand the putative function of StSOS1-13 during the immune response against *FOX*, its ortholog gene (*NbSOS1-13*) was identified based on the highest sequence similarity for VIGS (**Supplementary Figure S2**) with the pTVR2-*PDS* as an inner reference. As shown in **Supplementary Figure S3**, compared to mock-treated wild type (WT), there was no obvious phenotypic change in the leaves of pTRV2 empty vector (EV) transformed plants. The leaves of pTRV2-*NbSOS1-13* transformed strain showed yellow or even white pigmentation, and apparent shrinkage. The results of the RT-qPCR analysis indicated that the transcript level of *NbSOS1-13* in *pTRV2-NbSOS1-13* transformed plants was significantly reduced.

**Figure 2:**
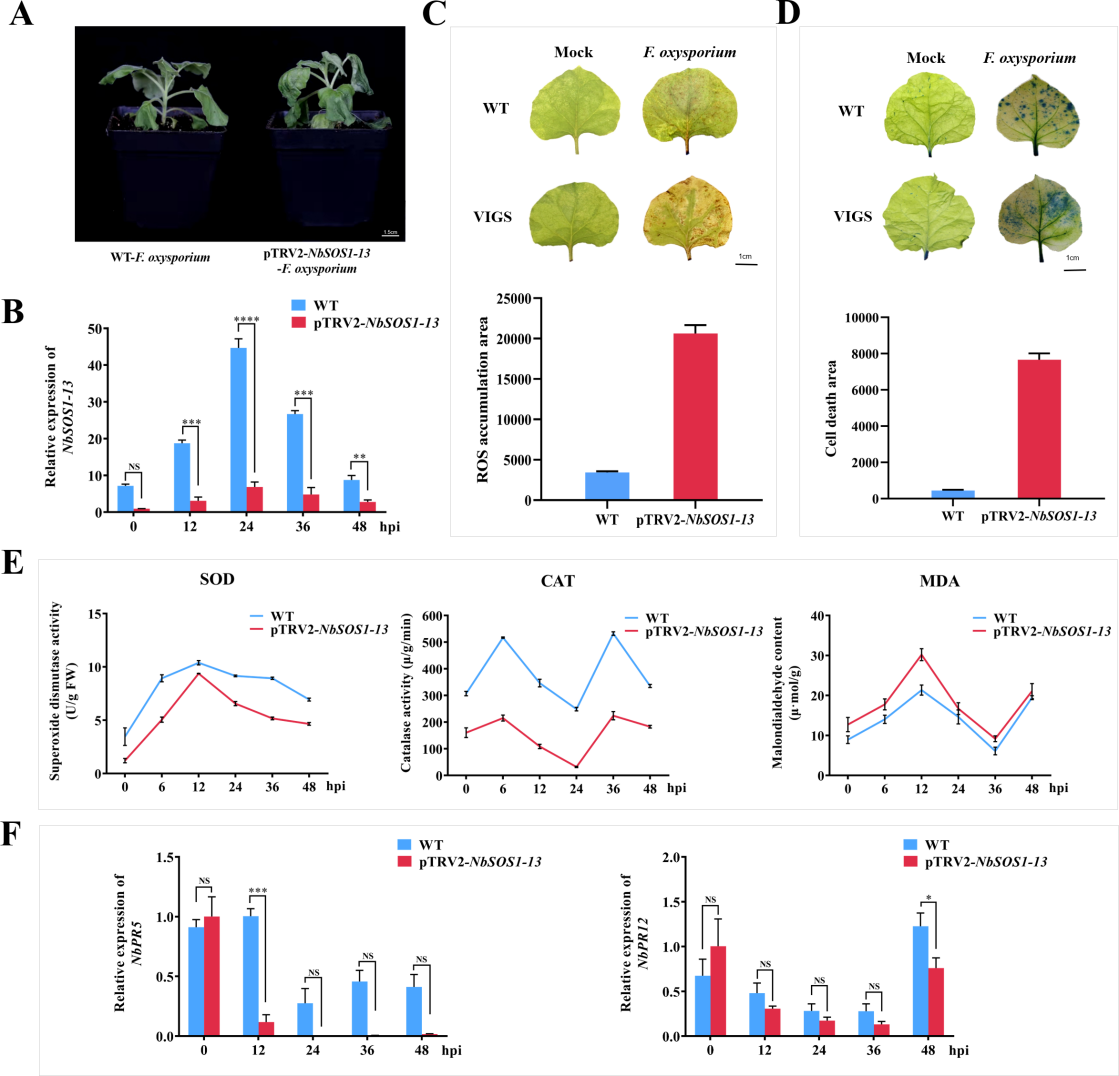
The resistance function analysis of the *NbSOS1-13* gene using VIGS. **(A)** The phenotypic responses of WT and *NbSOS1-13* VIGSed tobacco plants were observed following infection with *FOX*, with photographs captured five days post-inoculation; **(B)** The expression levels of WT and *NbSOS1-13* VIGSed tobacco plants infected with *FOX* were quantified using RT-qPCR at various time points post-infiltration; **(C)** The ROS were detected through DAB staining, with the ROS-positive areas quantified using ImageJ software; WT plants served as the control group; **(D)** Cell death in 4-week-old WT and *NbSOS1-13* VIGSed tobacco leaves was assessed two days post-inoculation with *FOX.* Trypan blue staining was employed to assess cell death, and ImageJ was utilized to quantify the regions of cell death, using WT as a control; **(E)** The activities of SOD and CAT, as well as the MDA content, were measured in WT and *NbSOS1-13* VIGSed tobacco leaves subjected to varying hpi with *FOX*; **(F)** RT-qPCR was conducted to evaluate the transcription levels of *NbPR5* and *NbPR12*, in WT and *NbSOS1-13* VIGSed tobacco plants following *FOX* treatment. Asterisks indicate significant differences based on two-way ANOVA (NS, no significant difference, *p<0.05, **p<0.01, ***p<0.001, ****p<0.0001), these experiments were performed with three independent biological replicates with similar results.

Within 60 hpi after infection, the vascular bundle browning and leaves wilting of *NbSOS1-13* VIGSed plants were faster and more severe than those of WT plants (**Figure 2A**). The transcription level of *NbSOS1-13* was significantly lower than that of WT leaves (**Figure 2B**) after continuous observation, especially at 24 h, it was only one fifth of that of WT. The staining degree of DAB and trypan blue in leaves was higher than that in WT plants, and the accumulation of ROS and the number of cell death were higher (**Figures 2C, D**). The activities of SOD and CAT decreased significantly, while the content of MDA increased significantly (**Figure 2E**). These results suggest that NbSOS1-13 may be involved in the early immune response of tobacco to *FOX*.

Furthermore, the expression of SA-responsible gene *PR5* and JA-responsible gene *PR12* related to the defense response (Anisimova et al., 2021) were compared between WT and *NbSOS1-13* VIGSed plants infected by *FOX*. The results showed that the expression levels of *NbPR5* and *NbPR12* were significantly lower in *NbSOS1-13* VIGSed plants than in WT plants at 12 h and 48 h, respectively (**Figure 2F**), suggesting that *NbSOS1-13* is positively correlated with gene expression of *NbPR5* and with *NbPR12.*

**Figure 3:**
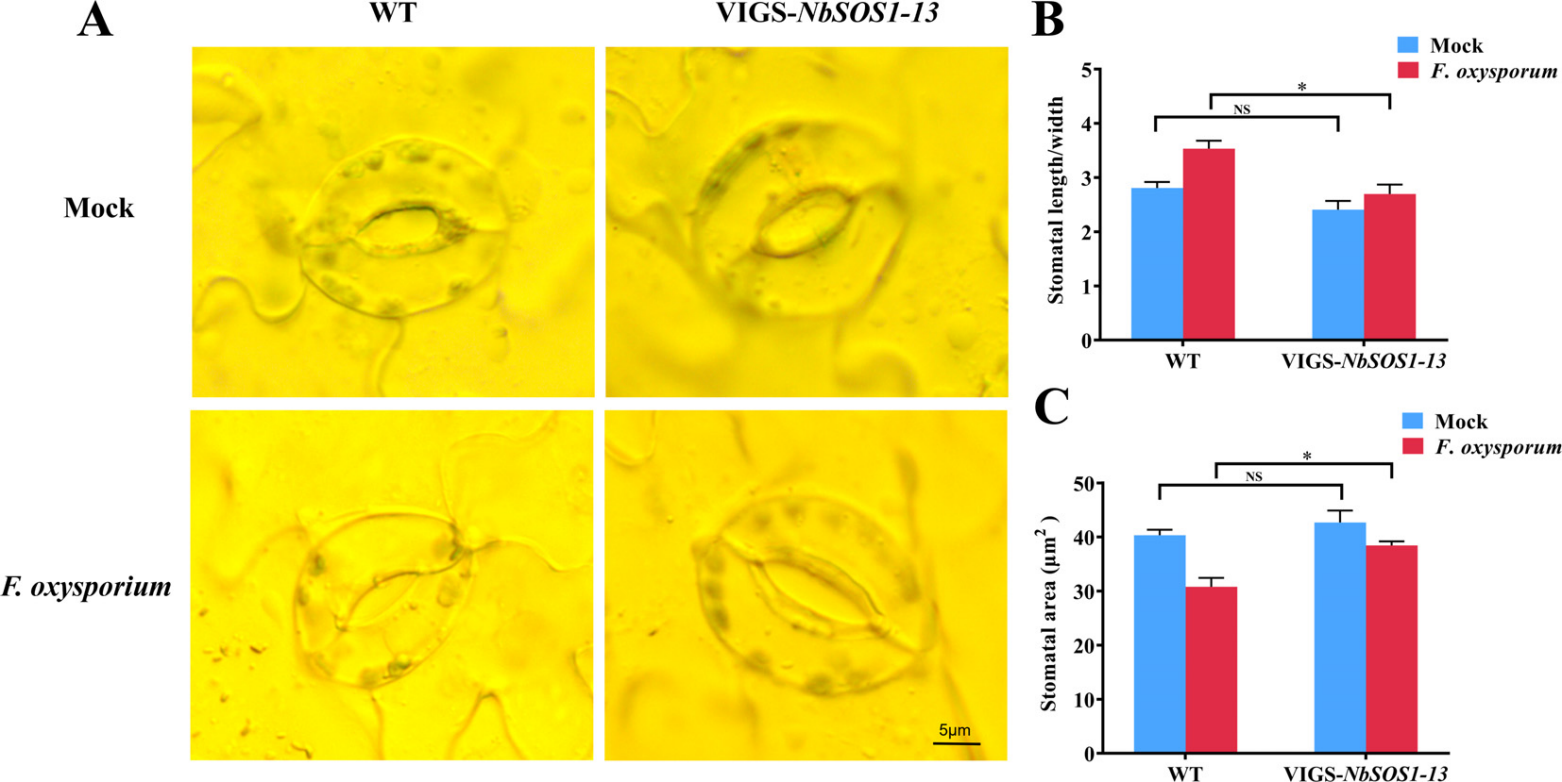
Stomata dynamics of tobacoo leaves under *FOX* treatment at 48 hpi. **(A)** Stomatal opening and closing of WT and *NbSOS1-13* VIGSed leaves at seeding stage under *FOX* treatment; **(B)** Aspect ratio of stomatal aperture in WT and *NbSOS1-13* VIGSed plants under *FOX* treatment 48 h calculated by ImageJ; **(C)** Leaf stomata area of WT and *NbSOS1-13* VIGSed plants under *FOX* treatment 48 h calculated by ImageJ. Asterisks indicate significant differences based on two-way ANOVA (NS, no significant difference, *p<0.05).

Based on the importance of stomatal movement in regulating transpiration in plants in response to adverse water conditions, the opening and closing of the leaf stomata pores of WT, *NbSOS1-13* VIGSed plants before and after infected with *FOX* were observed microscopically. The results showed no significant difference in leaf stomatal aperture between the *NbSOS1-13* VIGSed and WT plants before treated with *FOX*, but after treated with *FOX* for 48 hpi, compared with silenced plants, the leaf stomatal aperture of WT plants showed smaller (**Figure 3A**), the aspect ratio of stomatal aperture significantly greater (**Figure 3B**), and the stomata area significantly lesser (**Figure 3C**), respectively. It indicates that the WT plant shows a faster response to leaf stomatal movement under *FOX* treatment than the *NbSOS1-13* VIGSed plant.

### 3.3 Overexpression of *StSOS1-13* affected *Arabidopsis thaliana* against *FOX*

To elucidate the role of *StSOS1-13* gene in conferring resistance to *FOX*, *StSOS1-13* was heterologously overexpressed in Arabidopsis (**Supplementary Figure S4**). In Appendix F, two overexpressing lines OE5 and OE6 were obtained and were employed for resistance analysis, the RT-PCR showed that *StSOS1-13* was highly expressed compared with WT plants, and the root length was significantly increased at the seedling stage, but the plant height decreased significantly, and the florescence was delayed at the mature stage. These results indicated that the heterologous overexpression of *StSOS1-13* inhibited the growth of the aboveground part of Arabidopsis, but promoted the root elongation.

**Figure 4:**
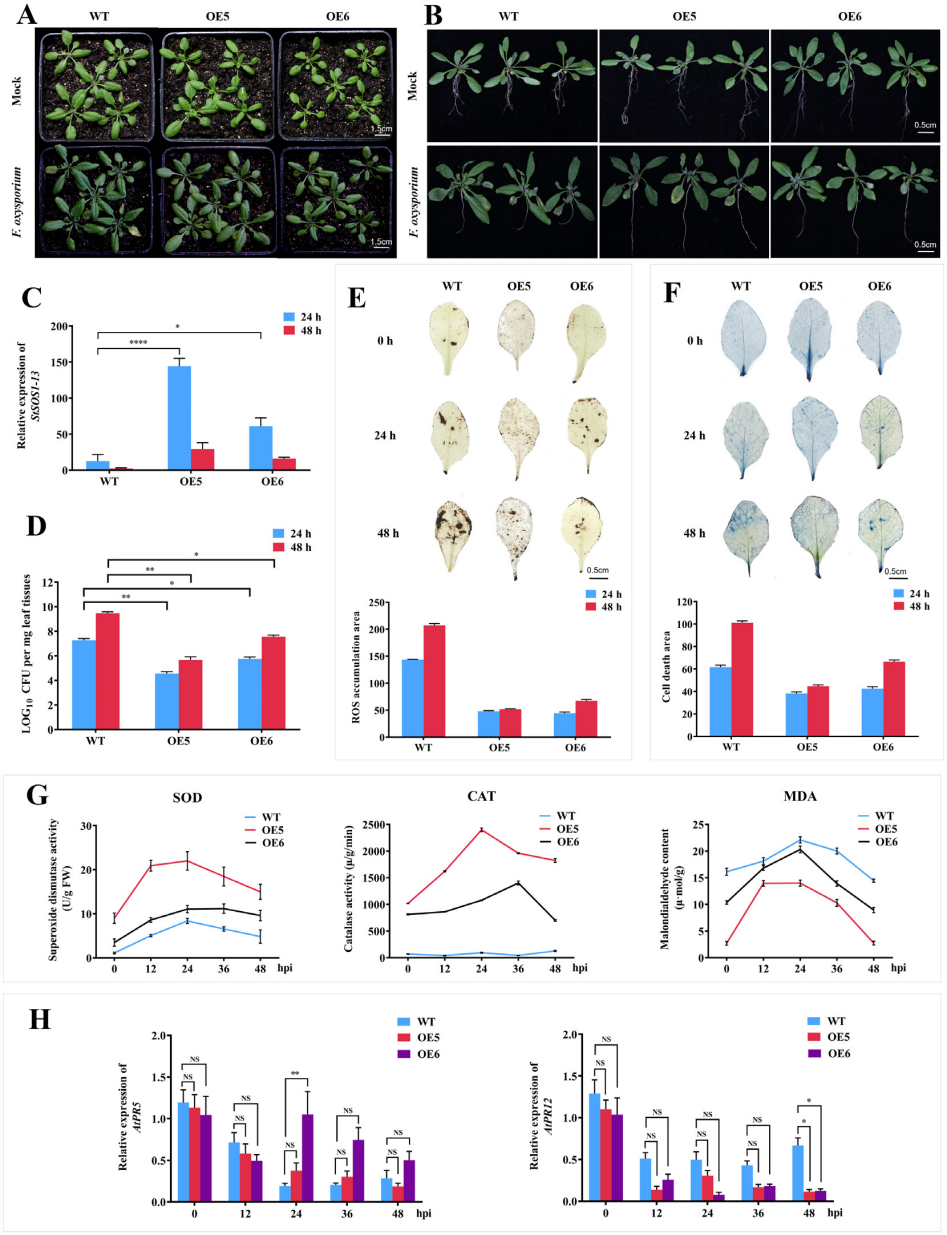
Heterologous overexpression of *StSOS1-13* impacts the resistance to *FOX* in Arabidopsis. **(A)** Effect of *FOX* treatments on growth of WT, OE5 and OE6 in Arabidopsis; **(B)** Effect of *FOX* treatment on root growth of WT and transgenic lines (OE5 and OE6); **(C)** Relative expression levels of *StSOS1-13* in WT, OE5 and OE6 inoculated with *FOX*; **(D)** Analysis of fungal biomass in the leaves of WT, OE5 and OE6 inoculated with *FOX*; **(E)** DAB staining of hydrogen peroxide (ROS) in the leaves of 2-week-old WT, OE5, and OE6 lines following inoculation with *FOX*; **(F)** Trypan blue staining indicates cell death in the leaves of 2-week-old WT, OE5, and OE6 lines post-inoculation with *FOX*; **(G)** Determination of SOD, CAT activities and MDA contents in leaves of WT, OE5 and OE6 inoculated with *FOX* for 0-48 hpi; **(H)** Transcription levels of *AtPR5* and *AtPR12*. Asterisks indicate significant differences based on two-way ANOVA (*p<0.05, **p<0.01, ***p<0.001), these experiments were performed with three independent biological replicates with similar results.

Three days after inoculation of *FOX* at seedling stage, compared with the symptoms of yellowing and wilting of most leaves of WT plants and obvious damage to root growth, OE5 and OE6 lines only showed slight yellowing or even no symptoms of disease (**Figure 4A**). The root growth was weakened, but the damage was mild (**Figure 4B**). The expression of *StSOS1-13* in WT, OE5 and OE6 lines increased after 24 h treatment, and the expression level of *StSOS1-13* in OE5 and OE6 lines was 11 and 5 times higher than that in WT, respectively (**Figure 4C**). The content of *FOX* in the leaves of OE5 and OE6 strains was significantly lower than that of WT lines from 24 h to 48 h, and decreased by 1.64 and 1.25 times compared with WT plants at 48 h (**Figure 4D**), respectively. The ROS accumulation, cell death number and the content of MDA in the leaves of OE5 and OE6 lines were significantly lower than those of WT plants (**Figures 4E–G**), while the activities of SOD and CAT were significantly increased, which were 25.8 and 11.6 times higher than those of WT plants at 24 hpi (**Figure 4G**). In the above results, compared with WT, the change of OE5 was more obvious than that of OE6, which was consistent with the higher overexpression level of OE5 than OE6 (**Supplementary Figure S5**). The above results showed that the heterologous overexpression of *StSOS1-13* could improve the disease resistance of Arabidopsis by promoting root growth and development, reducing the root damage caused by *FOX*, increasing the activity of antioxidant enzymes, eliminating ROS and reducing the damage caused by MDA production, and the level of heterologous overexpression was positively correlated with the disease resistance of Arabidopsis.

In contrast to the *NbSOS1-13* VIGSed tobacco plants, infection with *FOX* resulted in elevated expression levels of *AtPR5* in the OE5 and OE6 lines compared to WT plants at 24 hpi, with a particularly significant increase observed in the OE6 line. Unexpectedly, *AtPR12* expression levels were significantly reduced in the OE5 and OE6 lines compared to WT plants at 48 hpi (**Figure 4H**). These findings suggest that *StSOS1-13* may be positively correlated with the expression of the SA-responsible gene *AtPR5*, while exhibiting a negative correlation with JA-responsible gene *AtPR12*.

**Figure 5:**
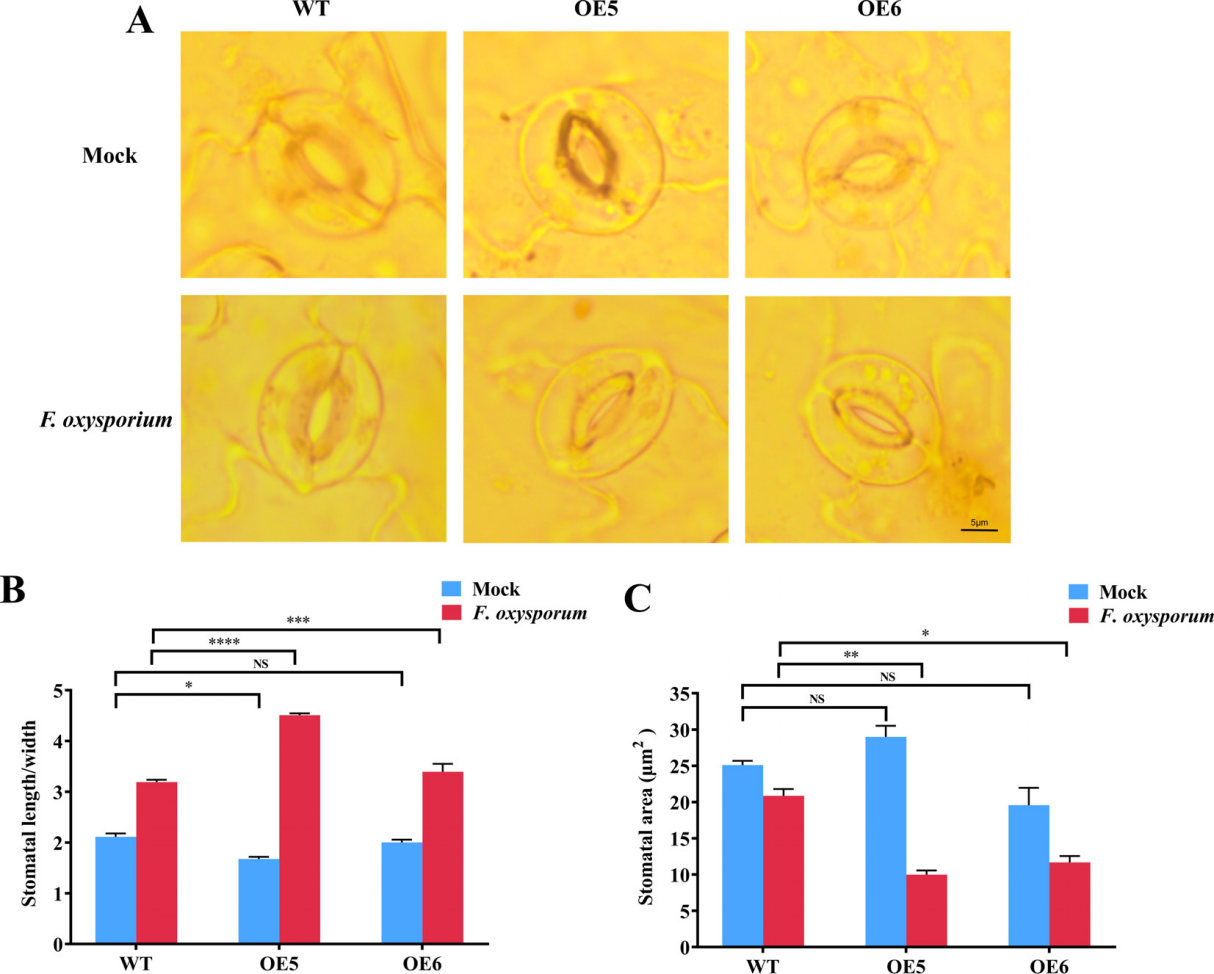
Stomata dynamics of Arabidopsis leaves under *FOX* treatment at 48 hpi. **(A)** Stomata aperture of WT and OE lines with mock (upper panel) and *FOX* (lower panel) treatment at 48 hpi; **(B)** Stomata aspect ratios (length/width) in WT, OE5 and OE6 lines before and after *FOX* treatment at 48 hpi calculated with ImageJ; **(C)** Leaf stomata area of WT, OE5 and OE6 lines before and after *FOX* treatment at 48 hpi calculated with ImageJ. Asterisks indicate significant differences based on two-way ANOVA (NS, no significant difference, *p<0.05, **p<0.01. ***p<0.001, ****p<0.0001).

There was no significant difference in leaf stomatal aperture among the OE5, OE6 lines and WT before treated with *FOX*, but the difference was significant after *FOX* treatment for 48 hpi (**Figure 5A**), this was similar to *NbSOS1-13* silence in tobacco. Specifically, the stomatal aspect ratios (length/width) of the OE5, OE6 lines and WT lines increased to 2.7, 1.63 and 1.58 times of that before treated with *FOX*, respectively (**Figure 5B**), and the stomata area decreased (**Figure 5C**), that was, stomata were partially closed. Compared to WT plants, the stomata of the OE5 and OE6 lines were significantly closed, and the closure of the OE5 line was even more significant. The above results indicate that the OE5 and OE6 lines show a faster response to leaf stomatal movement under *FOX* treatment than the WT, and further confirm the hypothesis of the silence experiment, that is, *StSOS1-13* is involved in stomatal closure to maintain water and achieve disease resistance in Arabidopsis.

## 4 Discussion

As an Na^+^/H^+^ antiporter, SOS1 has been confirmed playing an important role in salt tolerance in plants. There was evidence to suggest that *BjSOS1* implicate in disease resistance to *Plasmodiophora Brassicae* (Cheng et al., 2019). In previous studies, we found that *StSOS1* is involved in potato resistance to salt stress, but whether StSOS1 is involved in resistance to disease has not been reported. Therefore, this paper presents a preliminary study of whether and how *StSOS1* is involved in resistance to *FOX*.

### 4.1 SOS1-13 was actively involved in immune resistance to *FOX* in potatoes

In this study, six *StSOS1* genes were randomly selected for RT-qPCR analysis to assess their expression levels before and after infection with *FOX*. The results demonstrated that the expression levels of these genes initially increased and subsequently decreased, with *StSOS1-13* exhibiting the most pronounced changes (**Figure S1**). To further investigate the role of *StSOS1-13*, we employed gene silencing of its ortholog, *NbSOS1-13*, in tobacco and heterologous overexpression of *StSOS1-13* in Arabidopsis. The findings suggest that StSOS1-13 plays a significant role in mediating the immune response of both tobacco and Arabidopsis to *FOX* infection.

### 4.2 StSOS1-13 was positively involved in ABA-mediated early plant immune events triggered by *FOX* inductors

Under drought stress, stomata are closed which are regulated by ABA-mediated signal regulation mechanisms, thereby reducing transpiration, water loss and (Mega et al., 2015) photosynthesis (Gagné-Bourque et al., 2016). Under bacteria stress, plants also has evolved defense mechanisms for stomata closure, or inhibited pathogen-mediated stomata reopen upon sensing PAMPs to actively prevent bacteria from entering plant leaves (Melotto et al., 2006). Previous study has found that overexpression of *AtGAP1* can reduce the size of the stomatal pore and thicken the mesophyll cell wall of Arabidopsis, forming a defense barrier that effectively limiting the entry of pathogens into the leaves (Cheng et al., 2022).

Research indicates that soybean chitinase enhances plant disease resistance and mitigates cell death by augmenting ROS accumulation and the activity of active oxygen scavenging enzymes (Zhang et al., 2016). Colletotrichum infection increased the accumulation of MDA, CAT, and SOD in cucumber resistant and susceptible lines. The resistant lines have lower MDA content and higher SOD and CAT activity compared to the sensitive lines (Yang et al., 2022). The resistance factor Pti4/5/6 may mediate the expression of *PR* genes regulated by SA- and ET/JA-, and Pti4 may play a role in the communication between these pathways. Over-expression of *Pti4/5/6* in tomato plants increased CAT activity, decreased MDA content, and enhanced resistance to pathogens (Wang et al., 2021).

Our previous study found that *StSOS1-13* was significantly up-regulated about 250 times at 1 d in leaves under ABA treatment (Liang et al., 2023), and in this study, compared with the wild type, over-expression of *StSOS1-13* in Arabidopsis plants reduce the size of the stomatal pore (**Figure 5A**), increased SOD and CAT activity (**Figure 4G**), decreased ROS and MDA content (**Figures 4E, G**), and enhanced resistance to *FOX* (**Figures 4D, F**). However, *NbSOS1-13* VIGSed tobacco lines demonstrated the contrary effect (**Figures 2A, C, E**). The findings suggest that *StSOS1-13* plays a positive role in ABA-mediated signaling pathways during the initial immune responses of plants. This involvement is characterized by the closure of stomata, which serves to reduce transpiration and conserve water, thereby mitigating the effects of blocked xylem vessels and impaired water transport in roots induced by *FOX* elicitors. Additionally, StSOS1-13 contributes to the enhancement of the plant’s antioxidant capacity.

### 4.3 StSOS1-13 may play a role in coordinating SA- and JA-mediated pathways

Overexpression of certain *PR* genes, including *PR5* and *PR12*, greatly increased the level of plant defensive response to various pathogens (Liu et al., 2022), and overexpression of *PR5* may active many defense genes in the SA or JA/ET signaling pathways (Liu et al., 2022; Yan et al., 2017). In *A. sativum*, expression of the *PR1*, *PR3*, and *PR5* genes was thought to be a positive marker of plant resistance to *FOX* f. sp (Anisimova et al., 2021), and *PR1*, *PR2*, and *PR5* were the marker genes induced by SA (Ali et al., 2022). In Arabidopsis, the *cad-C*/*cad-D* mutation negatively affected *PR1* and *PR5* expression after infection with *P. syringae* pv (Rong et al., 2016). Our prior study also corroborated these findings, and there was a significant upregulation of *StSOS1-13* gene expression following 1 to 5 days of SA treatment, with a pronounced peak on day 3 (Liang et al., 2023). In the present study, the findings demonstrated that, compared to WT plants, the expression level of *StSOS1-13* was significantly up-regulated in the OE5 and OE6 lines at 24 and 48 hpi with *FOX* (**Figure 4C**). Additionally, the expression level of *AtPR5* at 12 hpi was significantly higher in the OE6 lines than in the WT plants (**Figure 4H**). Unexpectedly, the expression level of *AtPR12* exhibited an opposite trend to that of *AtPR5*, being significantly lower at 48 hpi in the OE5 and OE6 lines compared to the WT (**Figure 4H**). The findings indicate that the overexpression of *StSOS1-13* potentially enhances the expression of the SA-responsible gene *AtPR5*, while concurrently reduces the expression of the JA-responsible gene *AtPR12*. This divergent outcome may be attributed to the antagonistic effects of SA (is pivotal in early PTI) and JA (is essential in late ETI) signaling pathways during immune responses (Ali et al., 2018; Luo et al., 2020).

Conversely, in comparison to WT plants, the expression level of *NbSOS1-13* was significantly downregulated in *NbSOS1-13*-silenced tobacco lines between 24 and 48 hpi (**Figure 2B**), particularly at 24 hpi. Furthermore, the expression levels of *NbPR5* and *NbPR12* in the *NbSOS1-13*-silenced tobacco lines were significantly lower than those in the WT at 12 and 48 hpi with *FOX*, respectively (**Figure 2F**). Compared with the OE5 and OE6 lines, the consistency of *NbPR5* and *NbPR12* gene expression trends in *NBSOS1-13*-silenced tobacco may be due to the fact that the silencing of *NbSOS1-13* leads tobacco to enter the ETI stage from PTI more quickly, and there is a synergistic effect between SA and JA in this stage.

Based on the above analysis, it is speculated that StSOS1-13 plays a role in coordinating SA- and JA-mediated pathways, specifically, influencing *PR5* and *PR12* expression in response to *FOX* invasion during the early stages of immune events.

### 4.4 Ectopic overexpression of *StSOS1-13* enhances root development and inhibiting aboveground growth in Arabidopsis

Gibberellic acid (GA), a phytohormone, plays a crucial role in regulating multiple facets of plant development and growth, including seed development and germination, stem and root growth, cell division, and the timing of flowering (Kwon and Paek, 2016). *StSOS1-13* gene expression increased significantly within 1 to 2 days of GA treatment (Liang et al., 2023). Compared with WT, *StSOS1-13* overexpression Arabidopsis (OE5 and OE6 lines) significantly increased root length (**Figure 4B**) and decreased sensitivity to *FOX* (**Figures 4A, B**), and during the mature stage, the OE5 and OE6 lines exhibited a significant decrease in plant height, a reduction in stem length, a delay in flowering (**Supplementary Figure S5**). These results indicated that the ectopic overexpression of *StSOS1-13* may enhance resistance to *FOX* by inhibiting the growth of the aboveground part of Arabidopsis, but promoted the root elongation through positively participating in GA-mediated growth and development metabolic pathways. The results suggest that StSOS1-13 may enhance resistance to *FOX* by actively participating in GA-mediated growth and developmental metabolic pathways. This process promotes root development while inhibiting aerial growth in Arabidopsis.

### 4.5 StSOS1-13 acts as a key hub in various hormone signaling pathways and is involved in early plant immunity

Based on our findings and others existing studies (Dai et al., 2018; Feki et al., 2016; Guo et al., 2004; Kumari et al., 2017), a potential mechanism by which StSOS1-13 contributes to disease resistance in potato can be inferred as follows: upon infection by *FOX*, plants experience competition for essential nutrients and water with the pathogen, leading to water loss in plant tissues, elevated intracellular Na^+^ concentrations, Ca^2+^ influx, and the activation of downstream Ca^2+^ signal pathways. The proteins StSOS2 and StSOS3 interact to form a complex that activates StSOS1-13 (D et al., 2008; Yuan and Poovaiah, 2022), which plays a crucial role in pathogen immunity through signaling pathways mediated by ABA, GA, SA and JA. StSOS1-13 is actively involved in the early ABA-mediated PTI signaling pathway during the initial stages of *FOX* infection. This involvement includes enhancing the antioxidant capacity to mitigate damage caused by ROS accumulation, which further facilitates the binding of StSOS1-13 with the StSOS2-StSOS3 complex, and promoting stomatal closure to counteract the clogging of woody tubes and water loss (Gu et al., 2022) associated with the proliferation of *FOX*. In this study, plants overexpressing *StSOS1-13* exhibited slower growth and delayed flowering in their above-ground parts (**Supplementary Figure S5E**), potentially due to excessive stomatal closure (**Figure 5A**). This condition likely resulted in reduced photosynthesis and a deceleration of anabolic metabolism. The involvement of StSOS1-13 in the ABA-mediated signaling pathway appears to inhibit growth and development in the aerial portions of the plants (**Supplementary Figure S5E**), and slowed down *FOX* reproduction (**Figure 4D**). Conversely, overexpression of *StSOS1-13* is associated with increased GA-mediated root length (**Figure 4B**), which may enhance water absorption during stomatal closure (Gu et al., 2022) and provide resistance against *FOX* through root exudates (Lu et al., 2022; Zhang et al., 2020). Furthermore, the silencing or overexpression of *SOS1-13* was found to respectively changed the expression of *PR5* and *PR12*, suggesting that StSOS1-13 is involved in regulating SA and JA mediated immune responses. In general, StSOS1-13, as a regulate hub of the defense response to *FOX*, plays an indispensable role in multiple signaling pathways, thereby resisting the invasion of *FOX*. This study offers a novel perspective for further elucidating the mechanisms of StSOS1-13 mediated resistance to plant diseases.

However, The precise function of StSOS1-13 in the potato immune response requires more in-depth research, such as through genome editing techniques in potato to understand the function of this gene and its upstream and downstream regulatory relationships, and then use StSOS1-13 as a possible candidate gene for disease resistance breeding in the future.

## 5 Conclusions

In summary, this study shows that StSOS1 -13 can serve as a connection point for multiple signaling pathways, such as ABA, SA, JA, and GA, by improving the antioxidant defense system, promotes root development to enhance water uptake, and closes leaf stomata to conserve water from evaporation, in response to FOX stress in potatoes.

## Data Availability

The original contributions presented in the study are included in the article/**Supplementary Material**. Further inquiries can be directed to the corresponding authors.

## Footnotes

1
https://www.ncbi.nlm.nih.gov/tools/primer-blast/.
