# Supplementary material for: Beyond salt tolerance: SOS1-13’s pivotal role in regulating the immune response to Fusarium oxysporum in Solanum phureja
Source: Front Plant Sci. 2025 Mar 6;16:1553348. doi: 10.3389/fpls.2025.1553348 (PMC11922900; doi:10.3389/fpls.2025.1553348)
Supplement: Supplementary file 1 [file DataSheet1.docx]

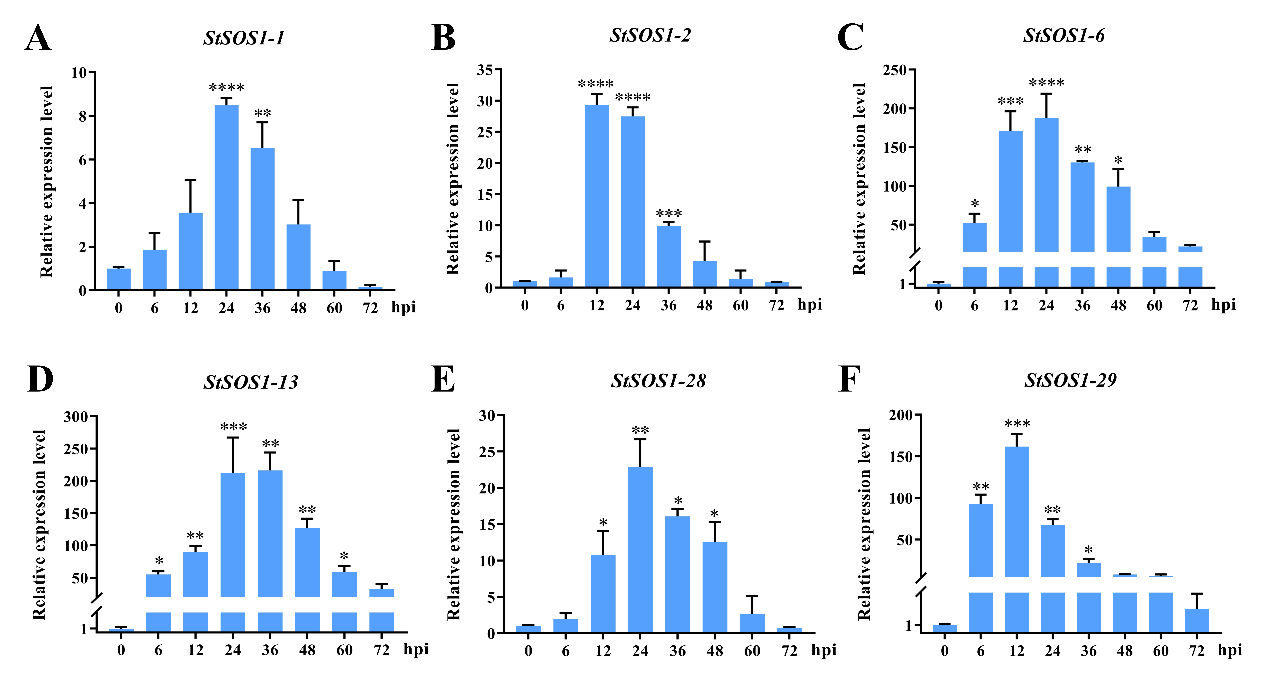


Figure S1. The expression anylysis of six *StSOS1* genes under *FOX* infection

(A-F) RT-qPCR anylysis of *StSOS1* genes in the leaves of potato plants under *FOX* infection.

The expression level of *StSOS1s* on control (at 0 h) was normalized as “1”. The vertical bars indicate the standard error of the mean. Asterisks indicate a significant difference based on the T test. (*, p<0.05, **, p<0.01, ***, p<0.001).
