# Supplementary material for: Beyond salt tolerance: SOS1-13’s pivotal role in regulating the immune response to Fusarium oxysporum in Solanum phureja
Source: Front Plant Sci. 2025 Mar 6;16:1553348. doi: 10.3389/fpls.2025.1553348 (PMC11922900; doi:10.3389/fpls.2025.1553348)
Supplement: Supplementary file 2 [file DataSheet2.docx]

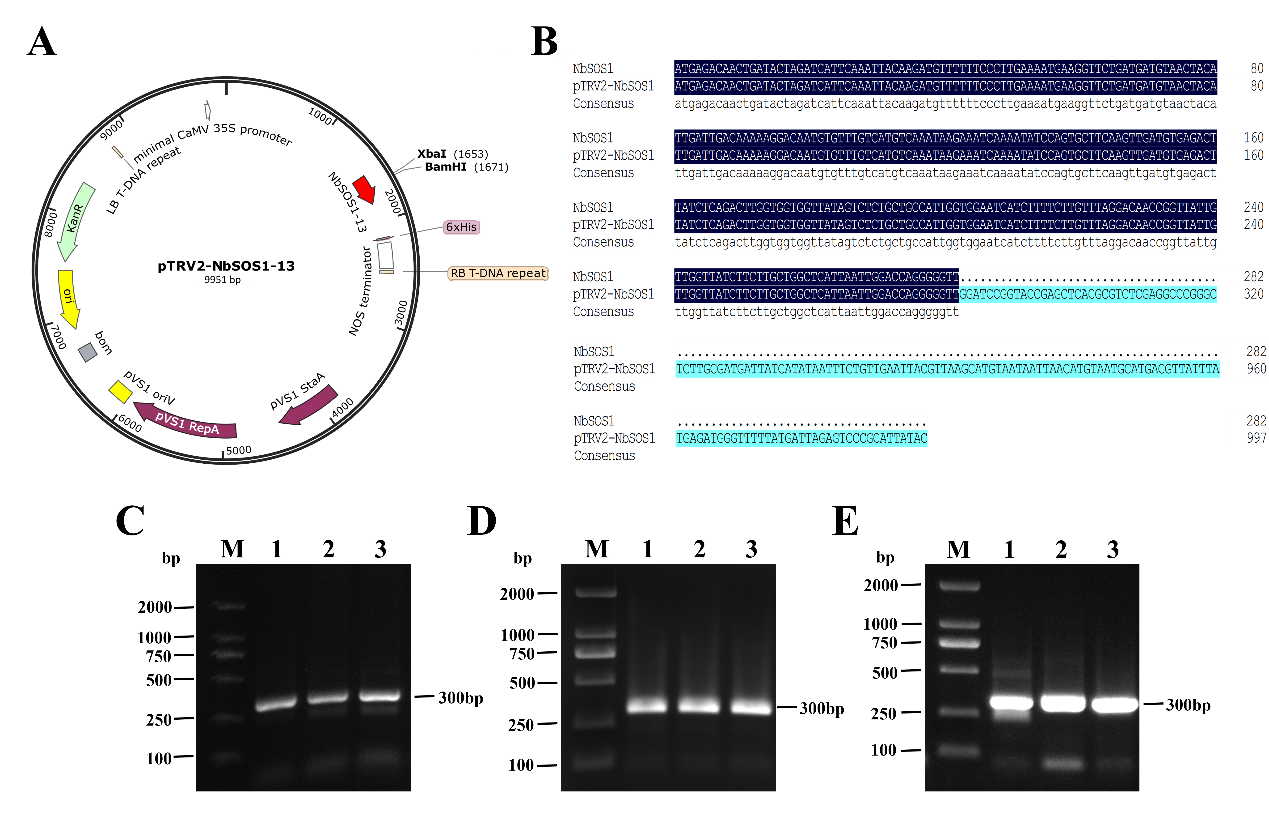


Figure S2. Construction of the pTRV2-*NbSOS1-13* vector for VIGS

(A) Schematic illustration of the construction of pTRV2-*NbSOS1-13* silencing clone;

(B) Sequencing results of pTRV2-*NbSOS1-13* vector;

(C) The electrophoretic images of *NbSOS1-13* interference fragment (300bp);

(D) The electrophoretic images of pTRV2-*NbSOS1-13* Escherichia coli colony PCR assay (1-3 swimming lanes were all successfully verified);

(E) The electrophoretic images of pTRV2-*NbSOS1-13* Agrobacterium colony PCR assay (1-3 swimming lanes were all successfully verified).

M: Trans2K® Plus DNA Marker
