# Supplementary material for: Beyond salt tolerance: SOS1-13’s pivotal role in regulating the immune response to Fusarium oxysporum in Solanum phureja
Source: Front Plant Sci. 2025 Mar 6;16:1553348. doi: 10.3389/fpls.2025.1553348 (PMC11922900; doi:10.3389/fpls.2025.1553348)
Supplement: Supplementary file 3 [file DataSheet3.docx]

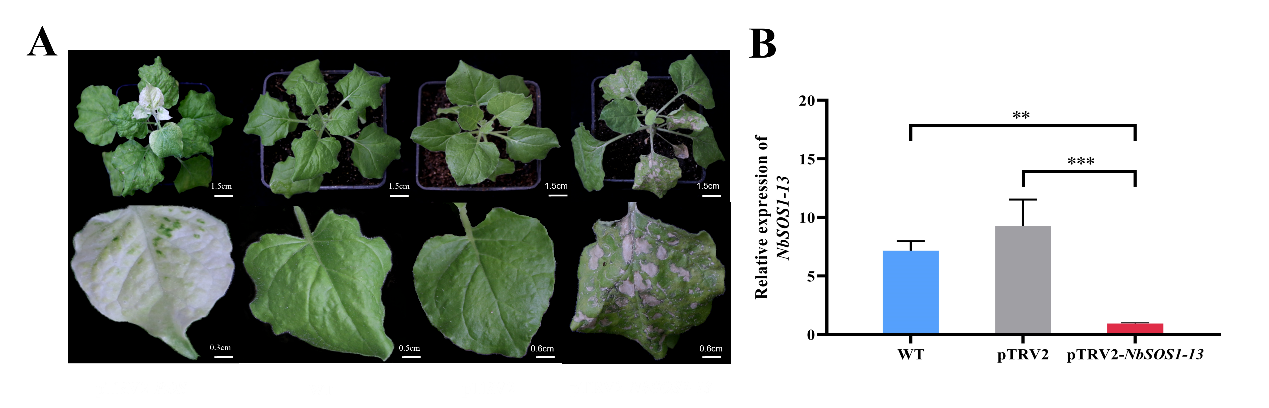


Figure S3. Silencing and RT-qPCR assessment of the *NbSOS1-13* gene

1. From left to right: positive control (pTRV2-*PDS*), blank control (WT), negative control (pTRV2) and silenced tobacco (pTRV2-*NbSOS1-13*);

(B) RT-qPCR analysis of *NbSOS1-13* transcript levels in the leaves of blank control (WT), negative control (pTRV2) and silenced tobacco (pTRV2-*NbSOS1-13*)
