# Supplementary material for: Beyond salt tolerance: SOS1-13’s pivotal role in regulating the immune response to Fusarium oxysporum in Solanum phureja
Source: Front Plant Sci. 2025 Mar 6;16:1553348. doi: 10.3389/fpls.2025.1553348 (PMC11922900; doi:10.3389/fpls.2025.1553348)
Supplement: Supplementary file 4 [file DataSheet4.docx]

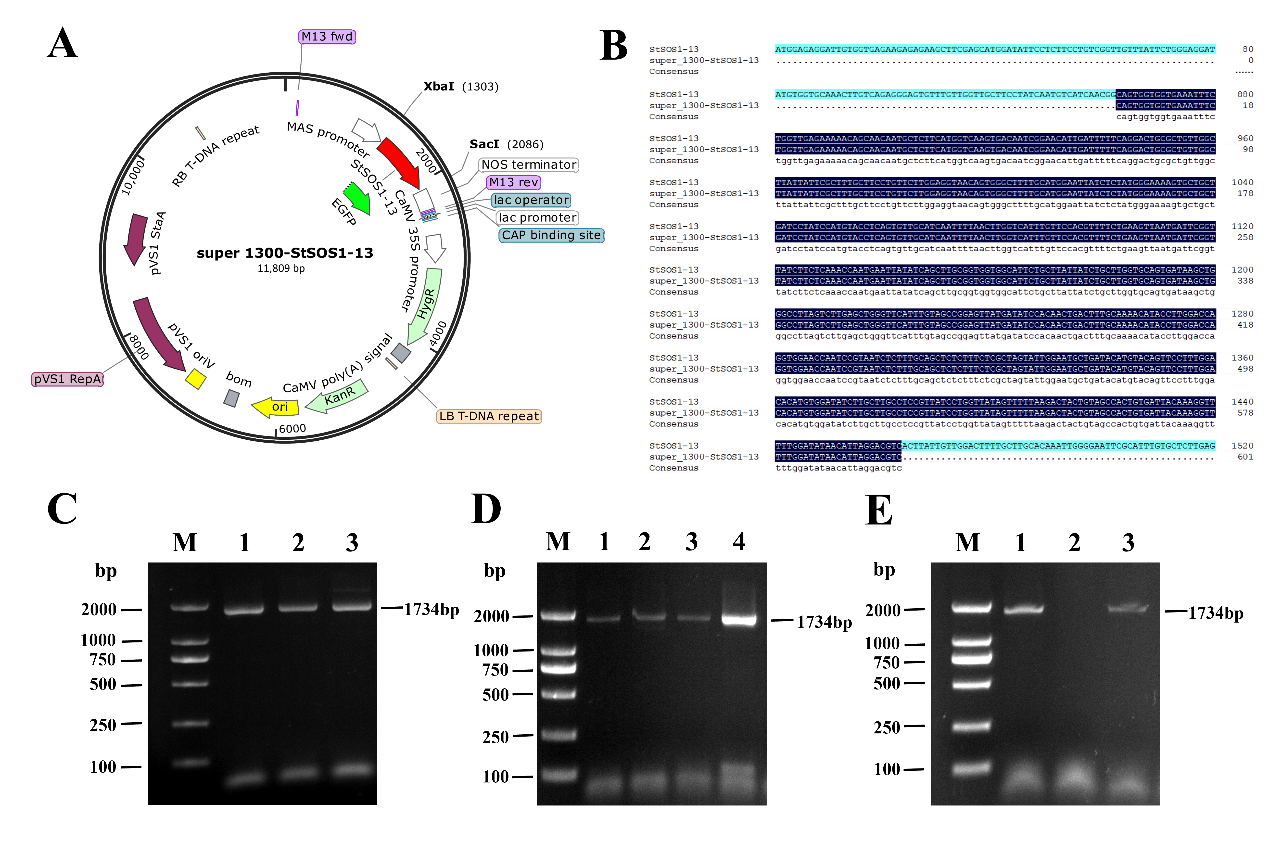


Figure S4. Construction of the vector for VOX

1. Schematic illustration of the construction of pCAMBIAsuper1300 and pCAMBIAsuper1300-*StSOS1-13* overexpression clone;

(B) Sequencing results of pCAMBIAsuper1300-*StSOS1-13* vector;

(C) The electrophoretic images of the full length pCAMBIAsuper1300-*StSOS1-13* fragment (1734bp);

(D) The electrophoretic images of pCAMBIAsuper1300-*StSOS1-13* *Escherichia coli* colony PCR assay (2-4 swimming lanes were all successfully verified);

(E) The electrophoretic images of pCAMBIAsuper1300-*StSOS1-13* *Agrobacterium* colony PCR assay (1, 3 swimming lanes were both successfully verified).

M: Trans2K® Plus DNA Marker
