# Supplementary material for: Beyond salt tolerance: SOS1-13’s pivotal role in regulating the immune response to Fusarium oxysporum in Solanum phureja
Source: Front Plant Sci. 2025 Mar 6;16:1553348. doi: 10.3389/fpls.2025.1553348 (PMC11922900; doi:10.3389/fpls.2025.1553348)
Supplement: Supplementary file 5 [file DataSheet5.docx]

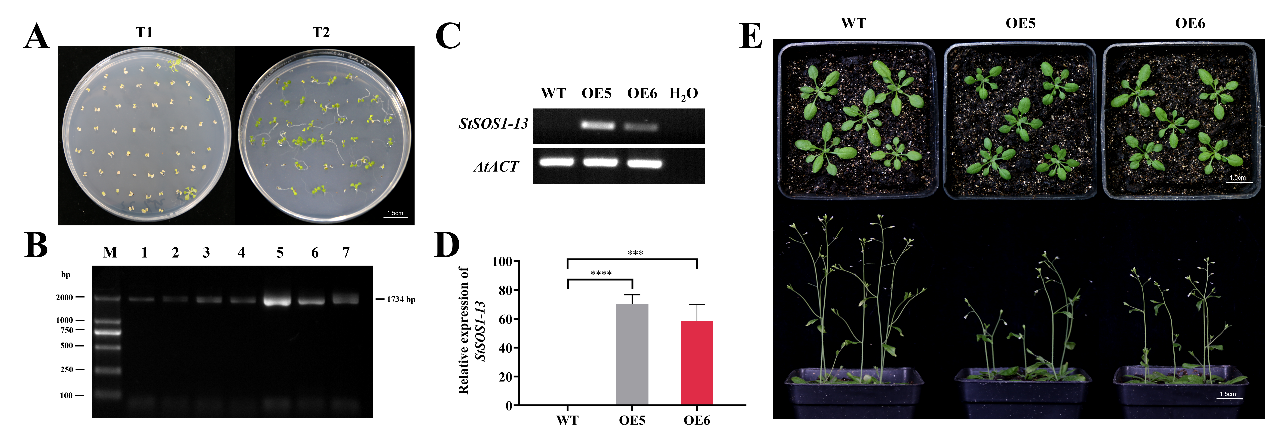


Figure S5. Heterologous overexpression of *StSOS1-13* in *Arabidopsis thaliana*

1. Arabidopsis seedling from first (T1) or second (T2) generation grown in MS medium on 25-50 mg/L hygromycin for 10 d;

(B) Confirmation of transgene integration into the Arabidopsis genome by PCR. (1-7 swimming lanes were all successfully verified). M: Trans2K^®^ Plus DNA Marker;

(C) RT-PCR analysis of the *StSOS1-13* in WT, heterologous overexpression Arabidopsis (OE5 and OE6) lines. *Actin* transcripts were used as cDNA internal controls;

(D) The expression analysis of *StSOS1-13* in WT, OE5, and OE6 by RT-qPCR;

(E) 4-week-old and 6-week-old plant phenotypes of wild-type and OE5 and OE6 lines.
