# Supplementary material for: Beyond salt tolerance: SOS1-13’s pivotal role in regulating the immune response to Fusarium oxysporum in Solanum phureja
Source: Front Plant Sci. 2025 Mar 6;16:1553348. doi: 10.3389/fpls.2025.1553348 (PMC11922900; doi:10.3389/fpls.2025.1553348)
Supplement: Supplementary file 6 [file DataSheet6.docx]

Note S1. >Phytoene dehydrogenase PDS CDS

ATGCCCCAAATCGGACTTGTATCTGCTGTTAATTTGAGAGTCCAAGGTAATTCAGCTTATCTTTGGAGCTCGAGGTCTTCGTTGGGAACTGAAAGTCAAGATGTTTGCTTGCAAAGGAATTTGTTATGTTTTGGTAGTAGCGACTCCATGGGGCATAAGTTAAGGATTCGTACTCCAAGTGCCACGACCCGAAGATTGACAAAGGACTTTAATCCTTTAAAGGTAGTCTGCATTGATTATCCAAGACCAGAGCTAGACAATACAGTTAACTATTTGGAGGCGGCGTTATTATCATCATCGTTTCGTACTTCCTCACGCCCAACTAAACCATTGGAGATTGTTATTGCTGGTGCAGGTTTGGGTGGTTTGTCTACAGCAAAATATCTGGCAGATGCTGGTCACAAACCGATATTGCTGGAGGCAAGAGATGTCCTAGGTGGGAAGGTAGCTGCATGGAAAGATGATGATGGAGATTGGTACGAGACTGGGTTGCACATATTCTTTGGGGCTTACCCAAATATGCAGAACCTGTTTGGAGAACTAGGGATTGATGATCGGTTGCAGTGGAAGGAACATTCAATGATATTTGCGATGCCTAACAAGCCAGGGGAGTTCAGCCGCTTTGATTTTCCTGAAGCTCTTCCTGCGCCATTAAATGGAATTTTGGCCATACTAAAGAACAACGAAATGCTTACGTGGCCCGAGAAAGTCAAATTTGCTATTGGACTCTTGCCAGCAATGCTTGGAGGGCAATCTTATGTTGAAGCTCAAGACGGTTTAAGTGTTAAGGACTGGATGAGAAAGCAAGGTGTGCCTGATAGGGTGACAGATGAGGTGTTCATTGCCATGTCAAAGGCACTTAACTTCATAAACCCTGACGAGCTTTCGATGCAGTGCATTTTGATTGCTTTGAACAGATTTCTTCAGGAGAAACATGGTTCAAAAATGGCCTTTTTAGATGGTAACCCTCCTGAGAGACTTTGCATGCCGATTGTGGAACATATTGAGTCAAAAGGTGGCCAAGTCAGACTAAACTCACGAATAAAAAAGATCGAGCTGAATGAGGATGGAAGTGTCAAATGTTTTATACTGAATAATGGCAGTACAATTAAAGGAGATGCTTTTGTGTTTGCCACTCCAGTGGATATCTTGAAGCTTCTTTTGCCTGAAGACTGGAAAGAGATCCCATATTTCCAAAAGTTGGAGAAGCTAGTGGGAGTTCCTGTGATAAATGTCCATATATGGTTTGACAGAAAACTGAAGAACACATCTGATAATCTGCTCTTCAGCAGAAGCCCGTTGCTCAGTGTGTACGCTGACATGTCTGTTACATGTAAGGAATATTACAACCCCAATCAGTCTATGTTGGAATTGGTATTTGCACCCGCAGAAGAGTGGATAAATCGTAGTGACTCAGAAATTATTGATGCTACAATGAAGGAACTAGCGAAGCTTTTCCCTGATGAAATTTCGGCAGATCAGAGCAAAGCAAAAATATTGAAGTATCATGTTGTCAAAACCCCAAGGTCTGTTTATAAAACTGTGCCAGGTTGTGAACCCTGTCGGCCCTTGCAAAGATCCCCTATAGAGGGTTTTTATTTAGCTGGTGACTACACGAAACAGAAGTACTTGGCTTCAATGGAAGGTGCTGTCTTATCAGGAAAGCTTTGTGCACAAGCTATTGTACAGGATTACGAGTTACTTCTTGGCCGGAGCCAGAAGATGTTGGCAGAAGCAAGCGTAGTTAGCATAGTGAACTAA
