# Supplementary material for: Beyond salt tolerance: SOS1-13’s pivotal role in regulating the immune response to Fusarium oxysporum in Solanum phureja
Source: Front Plant Sci. 2025 Mar 6;16:1553348. doi: 10.3389/fpls.2025.1553348 (PMC11922900; doi:10.3389/fpls.2025.1553348)
Supplement: Supplementary file 7 [file Table1.docx]

Table S1. The sequences of the primers used in the study

| **Primer** | **Sense sequence (5'to3')** | **Purpose** |
| --- | --- | --- |
| *Nbactin-F* | GCTCTAGAGGCAGTCACCTATTCCCTTG | RT-qPCR |
| *Nbactin-R* | CGGGATCCCAGGCAATAAGAGATTGCTA | RT-qPCR |
| *Stactin-F* | TATAACGAGCTTCGTGTTGCAC | RT-qPCR |
| *Stactin-R* | ACTGGCATACAGCGAAAGAACA | RT-qPCR |
| *Atactin-F* | AACCACTATGTTCTCAGGTATCGCT | RT-qPCR |
| *Atactin-R* | TGGACCTGCCTCATCATACTGC | RT-qPCR |
| *StSOS1-1-F* | GGATTGGCGATGGTATCCGT | RT-qPCR |
| *StSOS1-1-R* | CAATCTTGAGGGCAGCTCCA | RT-qPCR |
| *StSOS1-2-F* | TGTAAGCGACAGCCCACAAT | RT-qPCR |
| *StSOS1-2-R* | GAGTATGGCCTCCCCTGGTA | RT-qPCR |
| *StSOS1-6-F* | CTCTTGGAGGAGAACCGCTG | RT-qPCR |
| *StSOS1-6-R* | GGTACCAATGGCTCCGAACA | RT-qPCR |
| *StSOS1-13-F* | TCCTGGAGACGGTAGCCAAA | RT-qPCR |
| *StSOS1-13-F* | ATTCCACCAATGGCAGCAGA | RT-qPCR |
| *StSOS1-28-F* | ACAACCCGAGGCGAACTTAG | RT-qPCR |
| *StSOS1-28-R* | ACACTTGTCCCGGAAACCTC | RT-qPCR |
| *StSOS1-29-F* | CTCTAGGCCGCAGTGAGAAG | RT-qPCR |
| *StSOS1-29-R* | GGCCACTCCCATAAACACCA | RT-qPCR |
| *NbSOS1-13-F* | GCTCTAGATCAAGAAGAATGAGACACAACAGAC | Cloning |
| *NbSOS1-13-R* | CGGGATCCAACCCCCTGGTCCAATTAATGA | Cloning |
| *NbPR5-F* | TTCCTTCTTGCTTTTGTGAC | RT-qPCR |
| *NbPR5-R* | AAAGCGTATTCAGCTAAGCT | RT-qPCR |
| *NbPR12-F* | CCTTATGTTGATGCTGGTTT | RT-qPCR |
| *NbPR12-R* | AATAACAACTCCCTCCGATA | RT-qPCR |
| *VOX StSOS1-13-F* | GCTCTAGAATGGAGAGGATTGTGGTGAGA | Cloning |
| *VOX StSOS1-13-R* | CGAGCTCTTACAATACTCTGTTGTGCGTATCA | Cloning |
| *AtPR5-F* | CGGTACAAGTGAAGGTGCTCGTT | RT-qPCR |
| *AtPR5-R* | GCCTCGTAGATGGTTACAATGTCA | RT-qPCR |
| *AtPR12-F* | TCTACGCTGCTCTTGTTCTC | RT-qPCR |
| *AtPR12-R* | GAGCTGGGAAGACATAGTTG | RT-qPCR |
